# Poster Session I - A116 IMPROVING PEDIATRIC CELIAC DISEASE EDUCATION: OVERCOMING CHALLENGES THROUGH DIGITAL INNOVATION IN A CANADIAN SETTING

**DOI:** 10.1093/jcag/gwaf042.116

**Published:** 2026-02-13

**Authors:** M Lansing, D Issac, M Morrissey, M Chen, K Luick, J Wu, L Shirton, J Turner

**Affiliations:** Stollery Children’s Hospital, Edmonton, AB, Canada; Stollery Children’s Hospital, Edmonton, AB, Canada; Stollery Children’s Hospital, Edmonton, AB, Canada; Stollery Children’s Hospital, Edmonton, AB, Canada; Stollery Children’s Hospital, Edmonton, AB, Canada; Stollery Children’s Hospital, Edmonton, AB, Canada; Stollery Children’s Hospital, Edmonton, AB, Canada; Stollery Children’s Hospital, Edmonton, AB, Canada

## Abstract

**Background:**

Celiac disease (CD) is a common autoimmune disorder, where dietary exposure to gluten leads to small intestinal damage, altered immune function, and impaired nutrient absorption. Knowledge and commitment to maintain a life-long gluten free diet (GFD) is the only treatment. However, there remain many barriers to dietary adherence, including limited access to expert dietary counseling, difficulties understanding food labels, negative social impact of gluten free eating, and increased costs, amongst others. The current gold standard for dietary education is considered education with an expert dietitian. Dietetic services have become strained due to the rapidly rising prevalence of CD. Virtual education, increasingly used since the COVID-19 pandemic, is a potential resource to address barriers, such as limited access to expert dietetic care. A multimodal education approach is likely to be most effective, hence our team created online videos modules for newly diagnosed patients and their families.

**Aims:**

To evaluate the effectiveness of the video-based educational modules for caregivers of pediatric celiac disease (CD) patients under 18 years old.

**Methods:**

Four video modules were created by expert dietitians and reviewed by patient and physician stakeholders. Topics include understanding CD, label reading, cross-contamination prevention, healthy gluten-free eating, managing costs, and support resources. Consenting caregiver participants were recruited after a new celiac disease diagnosis of their child at Stollery Children’s Hospital. Educational videos were provided alongside standard dietary counseling. Participants completed pre- and post- Video test knowledge assessments and satisfaction surveys, along with demographic questionnaires, all administered via email using REDCap.

**Results:**

Overall, 18 caregivers completed the study: 95% Female, 47% held a bachelor’s degree, 30% had another household member with CD. Regarding education resources for CD and the GFD, 60% preferred to use all presented resources, 10% preferred 1:1 with a physician or dietitian, 5% preferred video online education, and 10% preferred both of the latter. Post-tests showed an improvement in overall test scores from 72% to 84% (p = 0.3) and a decrease in “Don’t know” responses from 52 to 13% (P = 0.01). Overall, 93.3% found the information useful and easy to understand, 86.7% would recommend the video to other caregivers.

**Conclusions:**

This study confirms that caregivers of newly diagnosed children with celiac disease valued a multimodal educational approach, including online videos that can be viewed at any convenient time. These videos were well received and significantly increased knowledge.

**Funding Agencies:**

WCHRI and MNCY for the videos

